# Supplementary material for: Collaborative intelligence in AI: Evaluating the performance of a council of AIs on the USMLE
Source: PLOS Digit Health. 2025 Oct 9;4(10):e0000787. doi: 10.1371/journal.pdig.0000787 (PMC12510544; doi:10.1371/journal.pdig.0000787)
Supplement: S2 File — (ZIP) [file pdig.0000787.s002.zip › S2 Data Raw data files containing unprocessed question inputs and LLM outputs including transcripts of deliberations/STEP3_Question-75-1_Type-MC-NJ.docx]

**Facilitator** **[Round 1]:** A 41-year-old woman comes to the emergency department because of a 3-day history of fever and a 2-day history of worsening flank pain with frequent and painful urination. She describes the pain as constant and says it worsens when she coughs or lies on her right side. She rates the pain as a 5 on a 10-point scale; ibuprofen has provided moderate relief of her pain. She also reports intermittent nausea but has not had vomiting or change in bowel habits. Medical history is significant for several uncomplicated urinary tract infections, most recently 8 months ago. Each infection resolved with antibiotic therapy. She currently takes no medications aside from her recent use of ibuprofen. She is sexually active with one male partner and uses condoms regularly. Vital signs are temperature 39.4°C (103.0°F), pulse 76/min, respirations 20/min, and blood pressure 128/74 mm Hg. Auscultation of the chest discloses normal S1 and S2. Abdomen is soft with normal bowel sounds. There is guarding on the right lateral side and tenderness to compression over the right costophrenic angle. Results of laboratory studies are shown: Serum: Urea nitrogen 18 mg/dL Creatinine 0.9 mg/dL Blood: Hemoglobin 12.8 g/dL WBC 20,000/mm3 Neutrophils, segmented 80% Lymphocytes 15% Eosinophils 2% Monocytes 3% Urine: Specific gravity 1.015 (N=1.003–1.029) Protein Negative Occult blood Negative Leukocyte esterase Positive WBC Too numerous to count Casts Occasional Urine and blood cultures are obtained and sent for analysis. Antibiotic therapy is initiated. Which of the following is the most appropriate next step? (A) Cystoscopy (B) Echocardiography (C) MRI of the abdomen (D) Renal ultrasonography (E) No additional study is indicated

**AI_A:** RESTATING THE QUESTION: The patient, a 41-year-old woman, has presented with symptoms of fever, right-sided flank pain that escalates with coughing or lying on her right side, increased frequency of urination, episodes of nausea, and multiple previous urinary tract infections. Lab results indicate high white blood cell count, both in the blood and urine, suggesting an infectious process. The medical team has already started her on antibiotic therapy and is now considering the best course of diagnostic action: cystoscopy, echocardiography, MRI of the abdomen, renal ultrasonography, or no further studies. PLANNING: Based on her symptoms and laboratory results, it's plausible to assume that she could be experiencing a renal issue, quite possibly a form of pyelonephritis, a type of kidney infection which occurs essentially when bacteria travel from the bladder and urethra up to the kidneys. The fact that she has a history of multiple urinary tract infections strengthens this hypothesis. However, it's still important to consider other assumptions such as appendicitis, nephrolithiasis (kidney stones), or other abdominal issue. Assessing those assumptions requires imaging of the kidneys which could be done via MRI or renal ultrasonography. CLINICAL REASONING: The use of MRIs in this context is not common unless there's a suspicion of complex kidney disease or tumor. A renal ultrasonography, however, is particularly useful in circumstances where acute pyelonephritis is suspected, as it helps determine whether there are any abscesses, obstructions or other structural abnormalities. Furthermore, it's non-invasive, quick, and relatively cheap, making it a favourable choice in this situation. Cystoscopy, and echocardiography don't directly apply here as they evaluate the bladder and heart respectively, which based on our working hypothesis doesn't align with our targeted anatomy. A decision against any further studies would represent a failure to adequately explore the cause of her symptoms, and thus wouldn't align with good medical practice. CONCLUDING: Considering her symptoms, past medical history and the available results from laboratory tests, the most logical step to pursue is renal ultrasonography. RESPONSE: Therefore, the most appropriate next step in this setting would be (D) Renal ultrasonography.

**AI_B:** RESTATING THE QUESTION: The patient is a 41-year-old female who has presented with symptoms of fever and severe flank pain, which exacerbate when she lies on her right side or coughs. She also has increased urinary frequency, reported as painful. Some relief of her symptoms is achieved using ibuprofen -- an NSAID used to alleviate pain. Her medical history marks recurrent urinary tract infections, but each was addressed successfully using antibiotics. Current lab findings reveal increased leukocyte count in both blood and urine samples, suggestive of an ongoing infection. The administration of antibiotic therapy has begun, and samples for urine and blood cultures are collected for further examination. We're being asked to determine the most suitable next step in her medical management. PLANNING: The assumptions include the patient's medical history and findings from labs, namely, the leukocytosis and the increasing severity of her symptoms, points toward a kidney-related infection. But should we consider her sexual history? Could it be gynecological rather than renal? These are pertinent questions, and I consider the former assumption should be given priority. CLINICAL REASONING(S): The patient's symptoms of fever, flank pain, and dysuria alongside a history of frequent UTIs suggest a possible pyelonephritis - an upper urinary tract infection affecting the kidneys. Typically present in females with a history of multiple lower urinary tract infections, pyelonephritis can present with all the symptoms listed. This diagnosis aligns with the clinical findings, including urinary leukocytes "too numerous to count," which are characteristic lab findings for pyelonephritis, suggesting a high level of infection within the urinary tract. The chief aim now should be to confirm this diagnosis and evaluate the extent of the infection. CONCLUDING: Considering the aforementioned clinical reasoning, the next appropriate step should be a diagnostic procedure that allows us to visualize the suspected area of pathology - the kidneys. RESPONSE: The most suitable next diagnostic step is (D) Renal ultrasonography - a non-invasive procedure that will allow visualization of the urinary tract, giving an instant picture of the kidneys to either confirm or reject our assumption of pyelonephritis, and inform treatment strategies. It can detect abnormalities like swelling of the kidney, indicative of infection, or other complications like abscess or obstruction that may necessitate different management steps.

**AI_D:** RESTATING THE QUESTION: So, our patient is a 41-year-old female who presented to the emergency department with complaints of fever, flank pain, and dysuria lasting for a few days. She has a past medical history of urinary tract infections. She currently shows signs of tenderness in the right flank area, and laboratory tests highlight an increase in white blood cells. Urine analysis also revealed the presence of many white blood cells, indicative of an infection. Considering her symptoms and her lab results, we need to decide what the next appropriate diagnosis step should be from the options provided, including cystoscopy, echocardiography, MRI of the abdomen, renal ultrasonography, or no additional study at all. PLANNING: Given her history of repeating UTIs and similar symptoms currently along with laboratory findings, the logical assumption to make would be that she has another UTI. This assumption could be incorrect due to the extended duration of the symptoms and their increased intensity, which could indicate a more severe infection that has ascended to the kidney, possibly pyelonephritis. This new assumption should now be prioritized for further investigation. CLINICAL REASONING: In terms of clinical reasoning, this patient's symptoms of flank pain, fever, and dysuria, elevated white blood cell count, and positive leukocyte esterase in the urine make the possibility of an uncomplicated UTI less likely. Instead, the symptoms and lab findings are more indicative of an upper urinary tract infection like pyelonephritis. Given the tenderness over the costophrenic angle (a possible sign of pyelonephritis) further investigations should be aimed at visualizing the kidney to confirm or rule out pyelonephritis. CONCLUDING: Considering her clinical presentation, past medical history, and current examination and lab results, the priority should be to evaluate for potential renal involvement, which would be reflected as pyelonephritis. This conclusion points us to use an imaging technique that can give us insights into renal structures. RESPONSE: Of the options provided, renal ultrasonography (choice D) would be the most appropriate next step. This non-invasive procedure offers the ability to visualize renal structures, identify any abnormality like abscesses or obstruction (e.g., due to stones) that may have led to the infection ascending to the kidney. On the other hand, cystoscopy (A) would mainly be useful to visualize the bladder and urethra, which isn't the primary concern here. Echocardiography (B) would be used if we suspected an associated cardiac issue, which isn't the case. An MRI of the abdomen (C) would be too broad and not specific to the kidneys. Finally, given the patient's symptoms and signs, doing no additional study (E) would not be clinically justifiable.

**AI_C:** RESTATING THE QUESTION: A 41-year-old woman is currently exhibiting symptoms including fever, flank pain, and frequent, painful urination. She has a history of uncomplicated urinary tract infections that were resolved with antibiotics. Her lab results indicate a high white blood cell count in both blood and urine, suggesting infection. Despite antibiotics being administered and samples sent for culture analysis, there is a need to determine further steps in her clinical management. The options given include cystoscopy, echocardiography, abdominal MRI, renal ultrasonography, or no further studies. PLANNING: Upon assessment, the woman's symptoms, past medical history, and laboratory results suggest urinary tract involvement, possibly extending towards the kidneys. The raised WBC count in blood and numerous WBC in urine point towards an ongoing infection. Given her history of recurrent UTIs, she could have developed pyelonephritis or an abscess. While cystoscopy, echocardiography, and MRI are useful investigations, considering the specific symptoms and clinical evidence, renal ultrasonography could be of immediate benefit. Let's evaluate this. CLINICAL REASONING: A urinary tract infection, when it travels upwards and affects the kidney, results in a condition called pyelonephritis, which presents with fever, flank pain, and urinary symptoms as seen in this patient's case. Her history of recurrent UTIs further substantiates this. A renal ultrasound, among the options provided, is a safe, non-invasive diagnostic modality that can evaluate the kidney structure, detect renal inflammation, abscess, or obstruction that might be causing her symptoms. Cystoscopy examines the lower urinary tract and doesn't provide much information about kidney involvement. Echocardiography is not relevant in this scenario unless there was clinical suspicion of endocarditis. Abdominal MRI provides detailed images but isn't typically the initial imaging choice due to cost, availability, and patient discomfort. CONCLUDING: Given the symptoms, lab findings, and past medical history, the suspicion of a kidney infection in this patient is high. This should be the focus of the next diagnostic step. Among the options offered, renal ultrasonography is a logical choice as it directly involves the possible source of the infection without posing unnecessary invasiveness or cost to the patient. Safety, efficacy, and relevance to her symptoms make it the most appropriate next step. RESPONSE: The most appropriate next step in managing this patient is to conduct a Renal ultrasonography (Option D).

**AI_E:** The question is about a 41-year-old woman with fever and right-sided flank pain. She has urinary symptoms and has had several urinary tract infections in the past. Her temperature, pulse, respiration, and blood pressure numbers show a fever. She exhibits soreness when the right side of her chest is compressed, particularly in the region of the costophrenic angle. Lab tests show elevated serum urea and high numbers of white blood cells, suggesting an infection. Her urine has a high specific gravity and is full of white blood cells, indicating infection in the urinary system. Upon the clinical manifestation, antibiotics are administered and cultures are sent for analysis. Now, the question is about the next appropriate step in her management—should it be cystoscopy, echocardiography, MRI of the abdomen, renal ultrasonography or no further action? Let's assume that the woman's symptoms and lab results suggest a severe urinary tract infection, possibly complicated by the involvement of her kidney—a condition known as pyelonephritis. Pyelonephritis is a urinary tract infection that extends to the kidney and is usually accompanied by systemic symptoms such as fever, shaking chills, and flank pain. We might also consider that the tenderness over her costophrenic angle implies pleuritic pain, suggesting the possible spread of infection to the pleura, the membrane surrounding the lungs. Current medical reasoning advises after starting empirical antibiotics and sending cultures, if a patient presents with symptoms suggesting pyelonephritis (fever, flank pain, leukocytosis, and bacteriuria), imaging is indicated to rule out complications such as abscess formation, hydronephrosis, or other obstructive uropathies, especially in severe or complicated cases. Thus my reasoning leads me to conclude that the most likely answer should be Renal ultrasonography (Choice D). Renal ultrasonography is non-invasive, readily available, and has a good sensitivity for detecting renal and perirenal abscesses, hydronephrosis, and stones. In contrast, cystoscopy and MRI of the abdomen can be more invasive or expensive, and echocardiography would be of little value in this context. Alternatively, no additional study [Choice E] would not be suitable, as we have hypothesised that identifying any complications should be a priority. In conclusion, the next appropriate clinical step would be to perform Renal ultrasonography to detect any presence of complications, such as renal or perirenal abscesses, hydronephrosis, or stones, which will help in appropriately managing and treating this patient's condition.

**Facilitator** The patient is a 41-year-old woman presenting with symptoms of fever, right-sided flank pain, increased frequency of urination, and a history of multiple urinary tract infections. Her lab results indicate a high white blood cell count in both blood and urine, suggesting an ongoing infection. Given her symptoms, past medical history, and laboratory results, it's plausible to assume that she could be experiencing a renal issue, possibly pyelonephritis, a type of kidney infection. This is further supported by her history of recurrent urinary tract infections. However, it's still important to consider other possibilities such as appendicitis, nephrolithiasis (kidney stones), or other abdominal issues. To assess these assumptions, imaging of the kidneys is required, which could be done via MRI or renal ultrasonography. The use of MRIs in this context is not common unless there's a suspicion of complex kidney disease or tumor. A renal ultrasonography, however, is particularly useful in circumstances where acute pyelonephritis is suspected, as it helps determine whether there are any abscesses, obstructions, or other structural abnormalities. Furthermore, it's non-invasive, quick, and relatively cheap, making it a favorable choice in this situation. Cystoscopy and echocardiography don't directly apply here as they evaluate the bladder and heart respectively, which based on our working hypothesis doesn't align with our targeted anatomy. A decision against any further studies would represent a failure to adequately explore the cause of her symptoms, and thus wouldn't align with good medical practice. Therefore, considering her symptoms, past medical history, and the available results from laboratory tests, the most logical step to pursue is renal ultrasonography. This non-invasive procedure offers the ability to visualize renal structures, identify any abnormality like abscesses or obstruction (e.g., due to stones) that may have led to the infection ascending to the kidney. This will help in appropriately managing and treating this patient's condition. Hence, the most appropriate next step in this setting would be (D) Renal ultrasonography.
